# Refugees’ views of the effectiveness of support provided by their host countries

**DOI:** 10.3402/ejpt.v3i0.8447

**Published:** 2012-10-24

**Authors:** Vito Zepinic, Maria Bogic, Stefan Priebe

**Affiliations:** Unit for Social and Community Psychiatry, Barts and The London School of Medicine and Dentistry, Queen Mary, University of London, London, UK

**Keywords:** War, trauma, refugees, support

## Abstract

**Background:**

The war in former Yugoslavia, which commenced in 1990, caused the biggest refugee crisis in Europe since World War II. There are numerous research investigations into the trauma and associated problems. However, there is no available publication concerning refugees’ own perception of the provided support in host countries.

**Aims:**

To investigate how refugees evaluated support received (helpful or detrimental) and what kinds of support they wish to receive in the future.

**Method:**

The study participants were 854 refugees from former Yugoslavia settled in the United Kingdom, Germany, and Italy. Alongside demographic data, they were assessed using *International Neuropsychiatric Interview (MINI), Life Stressor Checklist–Revised (LSC–R), Manchester Short Assessment of Quality of Life (MANSA), Matrix for Recording Health Care, Social Interventions (MACSI)*, and an open questions interview.

**Results:**

Data revealed that 99.3% of refugees received some kind of support. The most frequent support (98.7%) was primary health care and the least frequent (34.7%) was support in employment and further training. The most helpful (27.5%) was primary health care, and the most detrimental (11.6%) was legal support. The most desired types of support were help in employment (31.8%) and further education/training (20.5%). The educational level of refugees affected their perceptions of support as detrimental or desired.

**Conclusions:**

There are different levels of received and desired support among host countries. There are also differences in the perception of received and desired support with regard to the refugees’ educational levels.

Over the past two decades, the world has been witnessing the largest and most diverse migration ever recorded in history. According to the UNHCR, by the end of 2008 there were some 42 million forcibly displaced people worldwide and 15.2 million of them were refugees. In the early 1990s, the war in former Yugoslavia caused the biggest humanitarian crisis and migration in Europe since World War II: over 45% of the population from the war-affected territories were forced to leave their homes in internal or external migration (Zepinic, 1997, 2011). For several million people, the conflict was associated with various extremely stressful and traumatic experiences, including shelling, mass rape, imprisonment and torture, loss of family members, ethnic cleansing, and forced displacement (Agger & Mimica, 1996). They have undergone fearful and traumatic journeys as they were suddenly and forcefully removed from their home, unprepared, and without proper legal documentation. They may never obtain legal status and/or live without threat of deportation from their host country. Whilst most people affected by the war in former Yugoslavia stayed in the area of conflict being internally displaced, large numbers sought residence abroad in the hope of finding a solution for their plight: resettlement and integration in another country.

What refugees typically have in common is the painful loss of home and separation from loved ones. This may be associated with mixed emotions of sadness for what they have lost, as well as the elation for what they may have gained; the ambivalence of wanting to return and wanting to stay, and the long myriad ambiguities of living in two homes and two cultures (Falicov, 2005). On an individual level, the impact of forcefully and unwillingly leaving home and country can cause feelings of uncertain future and a sense of non-existence, powerlessness, and hopelessness. Refugees often end up in an unknown country where they know nobody, without contact with friends, relatives, or others from their community.

Although being lucky to escape, they feel lost and embody the proverbial expression “like a fish out of the water” or like “plant taken forcefully away from suitable place and planted into a desert with no water or shade” (common phrase in former Yugoslavia). Forceful departure from home and community causes disruption (internally) of one's state of self-continuity and (externally) with society at large. The nature and effectiveness of local resettlement and integration are difficult to measure in quantitative terms. Refugees commonly bring a range of vulnerability factors that host countries have to address. This includes separation from family, unemployment, foreign language proficiency, and lack of access to health and welfare services, which include difficulties with the refugee visa application process. There may be overwhelming feelings with the painful consequences of such a disrupting, disturbing, tragic, and hurtful experience.

Alongside support in accommodation, many refugees from former Yugoslavia have needed primary health care and also psychological intervention not only due to the war trauma suffered but also because of the experiences related to the settlement into a new unknown environment. There may be a cascade of events (war trauma and an uncertainty related to the new environment), resulting in an increase in symptoms of hyperarousal, greater recollection of the intrusive events, and therefore more avoidance of reminders—including helpers. While providing assistance, services should be aware of pre-existing risk factors that may influence the severity of maladaptive symptoms associated with the new environment; otherwise, the received support will achieve a limited result.

In this study, we aimed to investigate which health care and community-based interventions refugees from former Yugoslavia actually received and which interventions had been particularly helpful in their own view. Thus, the study set out to test the repeatedly stated but rarely empirically substantiated hypothesis, which is users prefer community-based interventions and social support designed to help them recover and successfully assimilate into a new society/community (Eastwood, 1998; Franciskovic et al., 2008; Williams & Westermeyer, 1986; Zepinic, 1997, 2008, 2010).

## Method

### Sampling techniques and participants

The study was conducted as a multi-centre epidemiological survey in eight countries. The rationale and methods have been described in detail elsewhere (Priebe et al., 2004). A combination of random and non-random sampling approaches was adopted to recruit refugees coming from the former Yugoslavia and currently residing in the United Kingdom, Germany, and Italy. In Germany and Italy, the potential interviewees were identified through local resident registers and snowball sampling. Potential participants on resident registers were sent a letter inviting participation and included up to two reminders. In the absence of accessible resident registers in the United Kingdom, the potential interviewees were contacted through community organisations and snowball sampling. In compliance with the United Kingdom's confidentiality law (Data Protection Act, 1998), community organisations mailed invitation letters and one reminder to their members without revealing individual names to the researchers.

Participants were included if they were born within the territory of former Yugoslavia; if they were between 18 and 65 years old; if they had experienced at least one war-related traumatic event; if they had experienced the last war-related event at or after 16 years of age; if they had no severe learning difficulty and no mental impairment due to a brain injury or other organic cause. The traumatic experience was established using a screening list containing 20 stressful events which people may have experienced during wartime (e.g., ethnic cleansing, shelling, sexual assault, and combat). People who had not been in the war-exposed areas during the war were not included in the study.

### Procedures and measures

All interviews were conducted face-to-face and, in line with participants’ preferences, interviews were carried out at their homes, community organisations or on the premises of the study research centres.

Participants’ age, gender, marital status, educational level, and employment status were obtained in a brief structured questionnaire. Potentially traumatic wartime experiences were assessed on an adapted version of the Life Stressor Checklist–Revised (LSC–R). The list is based on and similar to other methods used to assess trauma exposure (Wolfe & Kimerling, 1997). It assesses whether or not a participant had experienced any of the 24 potentially traumatic events during the war. Cumulative scores of trauma experiences were calculated.

Current mental disorders were assessed using the Mini International Neuropsychiatric Interview (MINI) (Sheenan, Lecrubier & Sheenan, 1998) which is a structured diagnostic interview assessing the symptom criteria used in the *Diagnostic and Statistical Manual of Mental Disorders IV* (American Psychiatric Association [APA], 1994). To assess a participant's degree of satisfaction with mental health, a single item from the Manchester Short Assessment of Quality of Life (MANSA) (Priebe, Huxley, Knight & Evans, 1999) was used. The item is subjective and satisfaction is to be rated on a 7-point Likert scale with a range between 1 = negative extreme and 7 = positive extreme of the scale. A single item question assessed whether a participant had taken medication for mental health difficulties since the war.

Utilisation of specific and non-specific health care and social interventions since the war was assessed using the Matrix for Recording Health Care and Social Interventions (MACSI), an instrument developed specifically for the present project. It is designed to collect data on nine major categories of health care and social interventions, including primary care, mental health care, specialist physical health care, housing support, employment support, leisure and social support, pensions and financial support, legal support, and support with information and advocacy. Within each category, the locally utilised interventions are recorded for each interviewed person so that a list of interventions within each category is built up locally based on what people utilised (bottom–up rather than top–down approach). As a consequence, an intervention that was available, but was not used by any of the participant does not feature in the results.

Subjective outcomes, treatment satisfaction, and wishes for further treatment were assessed using three open questions:

1. Which of the interventions you received have been helpful and, if there have been any, why and how have they helped?
2. Which of the interventions you received have been detrimental and, if there have been any, why and how have they harmed?
3. Which other interventions would you have wished to receive?

All those instruments for which there had been no validated translations in all languages were translated and then translated back into English. Out of the 11 interviewers, 9 were qualified psychologists, 1 was a sociologist, and 1 was an ethnologist. All researchers were bilingual (national language, plus Bosnian/Croatian/Serbian or Albanian) and participants were given the option to be interviewed in either their mother tongue or the host country's language. It should be noted that no problems occurred with regard to the nationalities of the participants and bilingual researchers.

Written informed consent was obtained from all participants prior to the interview. The study was approved by the relevant national ethics committees.

### Data analysis

Descriptive statistics were used to report socio-demographic and trauma related characteristics of the samples in each country. The prevalence rates of mental disorders were calculated as percentages of participants with a positive diagnosis. To analyse differences in socio-demographic characteristics, traumatic experiences, migration stressors, and prevalence of mental disorders between countries, χ^2^ tests were used for categorical variables and one-way analysis of variance for continuous variables.

Analyses were first conducted with variables from MACSI to identify the frequency of different types of received support since the war and subsequent migration. If the participant used at least one of the support/intervention from a particular group, it was categorised as “used” (for example, if they visited dentist, physician, or specialist, it was marked as “used primary health care” despite having few particular needed interventions).

Content analysis revealed eight main types of support identified by refugees as helpful, detrimental or wished for. The categories were as follows: primary health care, mental health care, accommodation support, social support, financial/material support, information/advocacy support, and legal help. The results are presented as frequencies of all registered services registered by MACSI, plus those that were nominated by refugees.

## Results

### Sample description

Overall, 21.2% of the participants responded to the invitation letters (United Kingdom = 22.6%, Germany = 26.5%, Italy = 14.5%), of which 47.6% (United Kingdom = 9.4%, Germany = 60.2%, Italy = 40.8%) did not meet the inclusion criteria and 52.9% (United Kingdom = 58.4%, Germany = 37.6%, Italy = 70.6%) were interviewed. For snowball sampling, response rates could not be established. In total, 854 (United Kingdom = 302, Germany = 255, Italy = 297) refugees were interviewed, of which 73.4% were recruited through data registers and community organisations.

Socio-demographic, trauma-related, and clinical characteristics of the samples are reported in Table 1.

**Table 1:** Description of the refugee samples in three countries

	Total (*N* = 854)	Germany (*N*=255)	Italy (*N*=297)	UK (*N*=302)	*p*
Female gender	438 (51.3)	133 (52.2)	137 (46.1)	168 (55.6)	0.063
Age (years), mean (SD)	41.6 (10.8)	41.9 (10.4)	38.9 (10.1)	43.9 (11.1)	<0.001
Country of origin					<0.001
Bosnia and Herzegovina	489 (57.3)	160 (62.7)	124 (41.8)	205 (67.9)	
Kosovo	150 (17.6)	34 (13.3)	45 (15.2)	71 (23.5)	
Serbia	108 (12.6)	38 (14.9)	66 (22.2)	4 (1.3)	
Croatia	84 (9.8)	19 (7.5)	46 (15.5)	19 (6.3)	
Macedonia	23 (2.7)	4 (1.6)	16 (5.4)	3 (1.0)	
Education level attained					0.232
None or primary	188 (22.0)	59 (23.1)	61 (20.5)	68 (22.5)	
Secondary	354 (41.5)	113 (44.3)	130 (43.8)	111 (36.8)	
Vocational/tertiary	312 (36.5)	83 (32.6)	106 (35.7)	123 (40.7)	
Married/cohabiting	652 (76.3)	189 (74.1)	242 (81.5)	221 (73.2)	0.035
Unemployed	469 (54.9)	175 (68.6)	85 (28.5)	209 (69.2)	<0.001
Count of war-related traumatic events, mean (SD)	6.8 (3.6)	7.8 (3.9)	5.2 (2.8)	7.4 (3.5)	<0.001
Any mental disorder	54.9	67.8	42.1	56.6	<0.001
Any mood disorder	42.7	56.3	30.0	44.0	<0.001
Any anxiety disorder	43.7	60.8	0.3	42.1	<0.001
Any substance abuse disorder	4.3	11.8	0.7	2.0	<0.001

The majority of the refugees came from Bosnia and Herzegovina (57.3%); 51.3% were women; the mean age was 42 years; 78% had completed secondary or higher education; 76.3% were married or cohabiting; and 54.9% were unemployed. The mean number of traumatic events experienced during the war was seven events. The three refugee samples significantly differed on all socio-demographic and trauma-related variables with the exception of gender and level of education (*p*<0.01 for all between-sample comparisons).

The majority (54.9%) of participants reported having at least one mental disorder, with anxiety (43.7%) and mood disorders (42.7%) being the most prevalent type of disorders. Substance use disorders were much less prevalent (4.3%). The prevalence rates of disorders showed statistically significant variation across countries. The prevalence estimates were frequently highest in Germany and lowest in Italy.

### Service use

Analyses were first conducted to identify the frequency of received support since the refugees arrived into their host countries. Data obtained through the interviews revealed that the war refugees from former Yugoslavia used a relatively wide range of social and health care support provided in the United Kingdom, Germany, and Italy. The frequency of basic categories of support used varied according to the type of service (Table 2). Primary health care, support with accommodation and financial or material support were the most used forms of help. However, there are differences in used services among countries in general, as well as among the categories of services provided.

**Table 2:** Frequency (%) of different types of support received by refugees since the war and migration

Type of support received	UK	Germany	Italy	Total
Any support received	296 (99.7)	250 (100.0)	292 (98.3)	838 (99.3)
Primary health care	293 (98.7)	251 (100)	290 (97.6)	834 (98.7)
Mental health care	109 (36.7)	164 (64.6)	38 (12.8)	311 (36.7)
Support in accommodation	274 (92.3)	211 (83.1)	114 (38.4)	599 (70.6)
Support in employment/training	123 (41.4)	113 (44.5)	58 (19.5)	294 (34.7)
Social support	181 (60.9)	103 (40.6)	14 (4.7)	298 (35.1)
Financial or material support	245 (82.5)	228 (89.8)	104 (35.0)	577 (68.0)
Legal support	199 (67.0)	107 (47.1)	42 (14.1)	348 (41.0)
Information and advocacy	127 (42.8)	128 (50.4)	84 (28.3)	339 (40.0)

The above table indicates a high proportion of received support by refugees from former Yugoslavia since their arrival at three European countries for resettlement. In general, the highest (98.7%) used support/intervention was primary health care (mostly primary and specialised health care) and the lowest was support with employment (34.7%). According to data in Table 2, in general, the proportion of the refugees receiving support/interventions was lower in Italy compared with the United Kingdom and Germany.

Despite many of the refugees categorised as highly traumatised individuals due to war experience, only 36.7% received support/intervention with regard to mental health problems. There was a wide difference in the usage of mental health services between countries (from 64.6% in Germany to 12.8% in Italy). Yet, mental health problems were common—based on the findings using the MINI. For example, according to data from the United Kingdom, 95.4% of refugees reported experiencing or witnessing an extreme traumatic event that included actual or threatened death or serious injuries to themselves or someone else, and 73.8% of them reported that since experiencing the traumatic event they re-experienced it in a distressing way such as maladaptive behaviour, nightmares, intense re-collections, flashbacks or psychosomatic reactions.

The data also revealed that 51.6% of the interviewed refugees reported symptoms of depression, and 44.0% reported general anxiety disorder symptoms. Furthermore, it was revealed that 41.4% of refugees were dissatisfied or displeased with their mental health condition and a considerable number (53.3%) of those reported taking psychotropic medication.

#### a) Which of the interventions you received have been helpful, why and how they helped?

The frequency of services used which the refugees found helpful is presented in Table 3. It is evident that the most helpful services were primary health care service (27.5%), financial or material support (25.7%), and support in accommodation (13.3%). It is interesting that support with regard to employment, further education, or training, as well as information and advocacy, were not found particularly helpful by the refugees in any country.

**Table 3:** The frequency (%) of services that were found helpful

Services used	UK	Germany	Italy	Total
Primary health care	67 (22.6)	141 (62.9)	17 (5.7)	225 (27.5)
Mental health care	31 (10.4)	78 (31.3)	3 (1.0)	112 (13.3)
Financial or material support	100 (33.7)	70 (27.9)	47 (15.8)	217 (25.7)
Support in accommodation	85 (28.6)	40 (15.9)	23 (7.7)	148 (17.5)
Legal support	36 (12.1)	29 (11.6)	13 (4.4)	78 (9.2)
Social support	4 (1.3)	11 (4.4)	2 (0.7)	17 (2.0)
All support helpful	19 (6.4)	5 (2.0)	3 (1.0)	27 (3.2)

Humanitarian programmes for war refugees are usually based on a psychosocial framework of proposed humanitarian interventions and emphasise community-collaboration, cultural adaptation, and mutual acceptance. Apart from the basic service already described, 5.0% of the refugees also found a language course helpful (9.2% in Germany), and 2% found community acceptance helpful.

#### b) Which of the interventions you received have been detrimental and, if there have been any, why and how have they harmed?

Among provided supports/interventions, the refugees found that the most detrimental (Table 4) were legal service (11.6%), support in accommodation (10.1%), and financial or material support (9.5%). Among the three host countries, provided supports/interventions in Germany were generally found to be more detrimental than in the United Kingdom or Italy.

**Table 4:** The frequency (%) of supports that were found detrimental

Services used	UK	Germany	Italy	Total
Legal support	31 (10.4)	58 (28.3)	9 (3.0)	98 (11.6)
Support in accommodation	6 (2.0)	75 (29.9)	4 (1.3)	85 (10.1)
Financial or material support	48 (16.2)	29 (11.6)	3 (1.0)	80 (9.5)
All support detrimental	9 (3.0)	20 (8.0)	3 (1.0)	32 (3.8)
Nothing detrimental	109 (36.7)	71 (28.3)	0 (0.0)	180 (21.3)

In Germany, a relatively high percentages of the refugees (29.9%) found support in accommodation quite detrimental. Upon their arrival in any country most refugees are taken to reception camps, in receiving countries often called reception centre, but refugees refer to them usually as “the camp”. These camps provide shelter, food, and water; however, according to refugees in Germany the organised accommodation was more expensive than private accommodation. It was common that several families unknown to each other shared rooms, bathroom, and kitchen. Hygiene in general was very poor and there was no privacy for the tenants. Many reported that in camp accommodation it was common for there to be a certain amount of stress arising from the use of alcohol or even drugs with no sanctions or any action taken by services that run the camps. Children were often exposed and witnessed a lot of violence and verbal abuse. Some refugees even stated that condition in the camp accommodation were even worse than in concentration camps where they had been imprisoned during the war.

In Germany, 28.3% of refugees also found the uncertainty about the legal status associated with constant fear of being deported quite detrimental. They did not have opportunity to travel even out of the town where they were accommodated. Some of them reported being unable to attend a funeral of a loved one, due to restrictions of travelling and that if they did not follow such strict restrictions, they would face immediate deportation. Some of the refugees found this quite inhuman and compared it with years of imprisonment, and reportedly suffered further deterioration of the mental health.

Regarding perceptions of financial or material support of being detrimental, refugees reported two quite embarrassing things: in Germany this was to do with the provision of support for clothing as a sort of uniform. In the United Kingdom, support for food was provided as vouchers. The refugees in Germany stated that in a few refugee centres they were supplied with the same colour and fashion of clothing. Effectively uniforms were quite recognisable in a humiliating way. Many of them even refused to go outside and mix with people in an attempt to avoid embarrassment due to the recognisable clothing.

In the United Kingdom, refugees found it quite embarrassing that vouchers were provided for food and that they were only able to use them in a particular food store. This meant that they were easily identified and what Summerfield (2001) has described as feeling “stigmatised”. Apparently due to public outrage these food vouchers were withdrawn after sometime. In general, it was also evident that in the United Kingdom, the level of financial support (social security payment) was found to be quite detrimental.

Despite these results concerning the adverse effects of specific supports, in general, a high percentage of the refugees found nothing detrimental: in the United Kingdom 36.7% and in Germany 28.3%.

However, the data revealed that prior educational achievement was associated with different perceptions of the detrimental effect of service provision. Only 30.5% of high and university-educated refugees reported that they had received non-detrimental support whereas 69.4% of the non-educated, primary or secondary educated refugees were of the same opinion. Of those who found that all received support has been detrimental, 59.4% were high or university educated. It is interesting that non-educated, primary or secondary educated refugees found primary health care (60.0%) and provided support in accommodation (64.7%) detrimental where 73.8% of those with high or university education found financial or material support quite detrimental.

#### c) Which other interventions would you have wished to receive and wish to receive in future?

The war refugees that are subject of this paper reported (Table 5) the following supports that they would have wished to receive and would wish to receive in the future:

**Table 5:** The frequency (%) of services that were wanted and are desired in future

Type of services	UK	Germany	Italy	Total
Primary health care	6 (2.0)	8 (3.2)	12 (4.0)	26 (3.1)
Mental health care	32 (10.8)	39 (15.5)	6 (2.0)	77 (9.1)
Further education/training	69 (23.2)	91 (36.3)	13 (4.4)	173 (20.5)
Employment	84 (28.3)	136 (54.2)	49 (16.5)	269 (31.8)
Recognition of education	53 (17.8)	34 (13.5)	15 (5.1)	102 (12.1)
Community acceptance	20 (6.7)	36 (14.3)	25 (8.4)	81 (9.6)
Financial or material support	20 (6.7)	23 (9.2)	83 (27.9)	126 (14.9)
Support in accommodation	22 (7.4)	7 (2.8)	83 (27.9)	112 (13.3)

These results (Table 5) indicate that, in general, among refugees the most desirable support is employment (31.8%) and further education/training (20.5%) to bring greater feelings of integration, independency, confidence, and recognition of self-values—instead of being overwhelmed by uncertainty about the future. Other supports that refugees’ desire are 14.9% financial or material support (27.9% in Italy) and 13.3% accommodation (27.9% in Italy and only 2.7% in Germany).

The data revealed that there were no differences in desired employment and primary health care regarding educational level between refugees. However, there were significant differences among other services desired. Non-educated, primary, or secondary educated refuges in a high percentage (73.8%) want financial or material support in future. On the other hand, those with high or university education did not express such a high percentage in desire of financial or material support (only 26.2%). However, a high percentage of them (87.3%) wanted recognition of their qualifications and 61.1% want further education. This indicates that educated refugees want to achieve a way for financial independence and make themselves active members of the society who are able to resume the everyday rhythms of life and re-establish a viable social and family identity (De Silva, McKenzie, Harpham & Huttly, 2005; Munoz, 1980; Summerfield, 2001; Weine et al., 2002).

## Conclusions

Forceful migration away from home and community by war is undoubtedly one of the most difficult forms of psychological distress and can affect an individual, group of people or even an entire nation. People affected by war are under high threat for their survival (existence), psychological health, and social position. Everything that was built for years—friendships, life targets, and values—is exposed to devastation and destruction. Individuals are horrified by the nonsense of the war devastations, disbeliefs and fears, and they are confronted with an identity crisis. War causes psychological traumas, which compel war victims to search for the vicinity of other people. On becoming refugees, many individuals feel ashamed and confused, withdrawn and not attuned, with a loss of self-continuity and self-cohesion. Beyond trauma, the war survivors have experienced the spectrum of psychological, social, and economic stresses of forced migration (Turkovic, Havens & Gregurek, 2004; Wilson, Friedman & Lindy, 2001).

For any war survivor the most desired action is to escape from the war-affected place and find a peaceful refuge to continue life. The arrival in another country as a refugee is accompanied by a first reaction of relief and gratitude due to escaping direct war destruction. However, this result can be of short-lived; life as a refugee is full of other psychological and interpersonal processes and conflicts. Many factors influence how agencies and carers who provide support/interventions understand the refugee's hopelessness, insecurity, devastation, and desperation. Subsequently, this will ultimately affect the refugee's ability to integrate successfully or not into a new community.

Upon their arrival into new country, the refugees need essentials, which they cannot take care of themselves (primary health care, accommodation, and financial support). It is a matter of judgement and timing to find a balance in organising support for them and also helping them to organise themselves. Our research revealed (Table 2) that in the host countries (United Kingdom, Germany, and Italy) they have received well-organised support (99.3% received some kind of support: 98.7% received primary health care, 70.6% received help in accommodation, and 68.0% received financial/material support), which helped them to settle in after escaping from the war-torn areas. In general, it seems that the best-organised support in basic needs was in the United Kingdom (98.7% refugees received primary health care, 92.3% accommodation, and 82.5% financial or material support). In Italy only 35.0% of the refugees received financial/material support and 38.4% support in accommodation. Subsequently, the refugees stated their level of satisfaction with received support: in Italy only 7.7% of them found provided accommodation was helpful and 5.7% primary health care, in comparison with Germany where 62.9% of the refugees found primary health care helpful.

In general, our data revealed that 3.8% of refugees found all provided support detrimental; however 21.3% stated that nothing was detrimental. The highest percentages of refugees who found nothing detrimental were in the United Kingdom (36.7%). In Germany 8.0% of refuges found all received support detrimental and 29.9% in particular found support in accommodation detrimental.

Our research revealed significant differences in perception of received support in regard to educational level. High and university educated refugees were more critical about the received supports: only 30.5% of them did not express complaints about support. However 69.4% of those with primary or secondary education stated that there was nothing detrimental. The interpretation about these results comes from an unstructured part of the interview (open questions) where high or university educated refugees reported that the financial support had been wasted and counterproductive making them inactive and unmoved in finding a long-term solution for the future. Financial support was welcomed upon their arrival to a new country by providing them with an opportunity to purchase the basic needed items (clothes, food, furniture, etc.). However, this “honeymoon” period did not exist for a long period of time, as highly educated refugees became the passive victims of their past relying on financial support. Some of them suggested that the financial support for over a decade made them totally dependent and it was a huge loss of time and intellectual potential in finding a better solution for integration into a new society.

The most desirable support among refugees in general was employment (31.8%), further education or training (20.5%), and financial/material help (14.9%). Primary and secondary educated refugees in a high majority (73.8%) desired financial/material support and 68.8% support in accommodation. On the other hand, high and university educated refugees desired recognition of their school degrees (87.3%) and 61.1% of them wanted further education or retraining. These data indicate that the refugees wanted support and interventions to help them to better integrate and blend into a new society as active survivors rather than as passive victims with stigmatisation.

The desire to become active members of a new society is supported by a naturalistic study from Sweden (Eastmond, 1998). Two groups of concentration camps survivors from Bosnia with similar socioeconomic and cultural background were by chance divided: half were sent to a place where they were provided with employment but no psychological support, and the other half were settled to a place where a full range of psychological services regarding trauma was available but refugees did not have an opportunity for employment. A follow-up study revealed clear differences between the two groups: those given work were doing better in adaptation and acceptance to a new society, whereas most of the second group was on indefinite sick leave. It is important to emphasise that although employment is generally positive for mental health, under-employment is a potential risk to psychological well-being (Aycon & Berry, 1996; Oliver, 2000).

In general, it seems that in spite of all efforts, the provided support failed to help refugees to take life gradually into their hands by stimulating them to take care of their own basic needs: restructuring and reorganising social interactions, to get active in daily activities, and re-establish self-confidence and self-respect. Thus it appears that it would be helpful to restructure the ways of support—targeting selective achievements over the longer term—rather than seeking general and temporary changes. This would improve the possibility of integration in a healthy way, in a reasonable time span and avoid factors that prevent integration for many years. A number of studies (Gorst-Unsworth & Goldenberg, 1998; Lie, 2002; Porter & Haslam, 2001, 2005; Steel, Silove, Bird, McGorry & Mohan, 1999) have noted that post-migration conditions and stressors in a new country, including separation from family, difficulties with refugee procedures or even detention, unemployment, and issue related to acculturation, may also exacerbate risk of the development and/or perpetuation of mental disorders among refugees.

A simple “trauma model” assumes a single causative relationship between pre-migration traumatic experience and subsequent impact of immigration on mental health. It is evident that social interventions and support in areas such as employment, further education, social support, and material assistance addressing these post-migration stresses may also be of a great benefit and may be a more preferred type of help for the population concerned (Summerfield, 2000).

Interventions in post-conflict reconstruction aim to facilitate the transition from war to peace and include a broad range of organised activities including “the social integration; community mobilisation; social integration of displaced persons (Wessels, 1999)”. How much these interventions are acceptable to refugees will also impact their mental health. Although our study was not primarily focused on refugees’ mental health, the data from MINI revealed that 51.6% of the interviewed refugees reported symptoms of chronic PTSD and depression, and 44.0% reported general anxiety. Furthermore, it was evident that 41.4% of refugees were dissatisfied or displeased with their mental health condition and there was a considerable number (53.3%) of those who reported taking psychotropic medication.

A study carried out by Franciskovic et al. (2008) found that 43.8% of a traumatised population in Croatia had clinically relevant symptoms of mental disorders 15 years after the war and that the organised systems of help to the war victims have neither been recognised enough nor have they fulfilled their objective as expected. A study (Zepinic, 2001) of 20 concentration camp survivors from Bosnia who arrived in Australia, found that all of them experienced high level of suicide tendencies as a result of social withdrawal, feeling estrangement from others, with low self-esteem and loss of confidence. It revealed that a lack of integration into new society caused “absence of all positive thinking, deciding not to want anything, and not trying to get something that was wanted”. The study also revealed that survivors believed they would not get better, nor solve personal problems, would have nothing to look forward to, would fail to achieve personal goals, and viewed the future with pessimism. This issue about integration should be investigated further in order to obtain a real picture of refugees’ mental health upon arrival to a host country and how received support and resettlement (post-migration factors) impacts their mental health.

This study, like any other, has its limitations that could be also used as indication for further research. By using only one item from MANSA to assess the quality of life considering potential and actual impact on refugees’ adaptation and integration into new society, we accept it is a narrow and limited approach to this important aspect of settlement. To fully explore such important area of new refugees’ life requires another study, or few studies. The results in this study, with all its limitations in small response rate and high ineligibility rate, has been carried out to produce evidence to indicate which steps should be taken to improve primary as well as mental health care and social adaptation to refugees who arrive in newly adopted countries. However, more detailed research about mental health and socio-economic problems among the refugees in each country would definitely answer many unanswered questions that we were unable to give in this study.

## References

[1] Agger I, Mimica J (1996). Psycho-social assistance to victims of war in Bosnia & Herzegovina and Croatia.

[2] American Psychiatric Association (1994). Diagnostic and statistical manual of mental disorders.

[3] Aycon Z, Berry J. W (1996). Impact of employment-related experiences on immigrants’ psychological well-being and adaptation to Canada. Canadian Journal of Behavioural Science.

[4] De Silva M. J, McKenzie K, Harpham T, Huttly S. R. A (2005). Social capital and mental illness: A systematic review. Journal of Epidemiological and Community Health.

[5] Eastmond M (1998). National discourses and the constructions of difference: Bosnian Muslim refugees in Sweden. Journal of Refugee Studies.

[6] Falicov C. J (2005). Therapeutic care for refugees: No place like home.

[7] Franciskovic T, Tovilovic Z, Sukovic Z, Stevanovic A, Ajdukovic D, Kraljevic R (2008). Health care and community-based Interventions for war-traumatised people of Croatia: Community based study of service use and mental health. Croat Medical Journal.

[8] Gorst-Unsworth C, Goldenberg E (1998). Psychological sequelae of torture and organised violence suffered by refugees from Iraq: Trauma-related factors compared with social factors in exile. British Journal of Psychiatry.

[9] Lie B (2002). A 3-year follow-up study of psychological functioning and general symptoms in settled refugees. Acta Psychiatrica Scandinavica.

[10] Munoz L (1980). Exile as bereavement: Social-psychological manifestations of Chilean exiles in Great Britain. British Journal of Medical Psychology.

[11] Oliver P (2000). Employment for professional migrants to New Zealand—barriers and opportunities.

[12] Porter M, Haslam N (2001). Forced displacement in Yugoslavia: A meta-analysis of psychological consequences and their moderators. Journal of Traumatic Stress.

[13] Porter M, Haslam N (2005). Predisplacement and postdisplacement factors associated with mental health of refugees and internally displaced persons. Journal of American Medical Association.

[14] Priebe S, Gavrilovic-Jankovic J, Schuetzwohl M, Galeazzi G. M, Lecic-Tosevski D, Ajdukovic D (2004). A study of long-term clinical and social outcomes after war experiences in ex-Yugoslavia – Methods of the “CONNECT” project. Psychiatry Today.

[15] Priebe S, Huxley P, Knight S, Evans S (1999). Application and results of the Manchester Short Assessment of Quality of life (MANSA). International Journal of Social Psychiatry.

[16] Sheenan D. V, Lecrubier Y, Sheenan K. H (1998). MINI–international neuropsychiatric interview: The development and validation of a structured diagnostic psychiatric interview for DSM-IV and ICD-10. Journal of Clinical Psychiatry.

[17] Steel Z, Silove D, Bird K, McGorry P, Mohan P (1999). Pathways from war trauma to posttraumatic stress symptoms among Tamil asylum seekers, refugees and immigrants. Journal of Traumatic Stress.

[18] Summerfield D (2000). War and mental health: A brief overview. British Medical Journal.

[19] Summerfield D (2001). Asylum-seekers, refugees and mental health services in the UK. Psychiatric Bulletin.

[20] Turkovic S, Havens J. H, Gregurek R, Wilson J. P (2004). Strengthening psychological health in war victims and refugees. Broken spirit.

[21] (1998). UK Parliament: Data Protection Act.

[22] Weine S. M, Danieli Y, Silove D, van Ommeren M, Fairbank J. A, Saul J (2002). Guidelines for international training in mental health and psychological interventions for trauma exposed populations in clinical and community settings. Psychiatry.

[23] Wessels M. G (1999). Systematic approaches to the understanding and prevention of genocide and mass killing. Journal of Peace Psychology.

[24] Williams C, Westermeyer J (1986). Refugee mental health in resettlement countries.

[25] Wilson J. P, Friedman M. J, Lindy J. D (2001). Treating psychological trauma and PTSD.

[26] Wolfe J, Kimerling R, Wilson J, Keane T. M (1997). Gender issues in the assessment of posttraumatic stress disorder. Assessing psychological trauma and PTSD.

[27] Zepinic V, Ferguson D, Barnes (1997). Psychosocial characteristics of war-related posttraumatic stress disorder (Chapter 10). Perspectives of transcultural mental health.

[28] Zepinic V, Raphael B, Malak A. E (2001). Suicidal risk with war-related posttraumatic stress disorder (Chapter 14). Diversity and mental health in challenging times.

[29] Zepinic V (2008). Healing traumatic memories: A case study. Dynamische Psychiatrie.

[30] Zepinic V (2010). Defining war-related complex trauma: Is this one impossible task?. International Journal of Health Science.

[31] Zepinic V (2011). Understanding and treating complex trauma.

